# Supplementary material for: The interactome of intact mitochondria by cross-linking mass spectrometry provides evidence for coexisting respiratory supercomplexes
Source: Mol Cell Proteomics. 2017 Dec 8;17(2):216–32. doi: 10.1074/mcp.RA117.000470 (PMC5795388; doi:10.1074/mcp.RA117.000470)
Supplement: Supplemental Data [file supp_RA117.000470_133922_0_supp_23978_fzffwf.zip › spectra_annotation/mito_spectra_annotation/141-1-9-1-10-1.pdf]

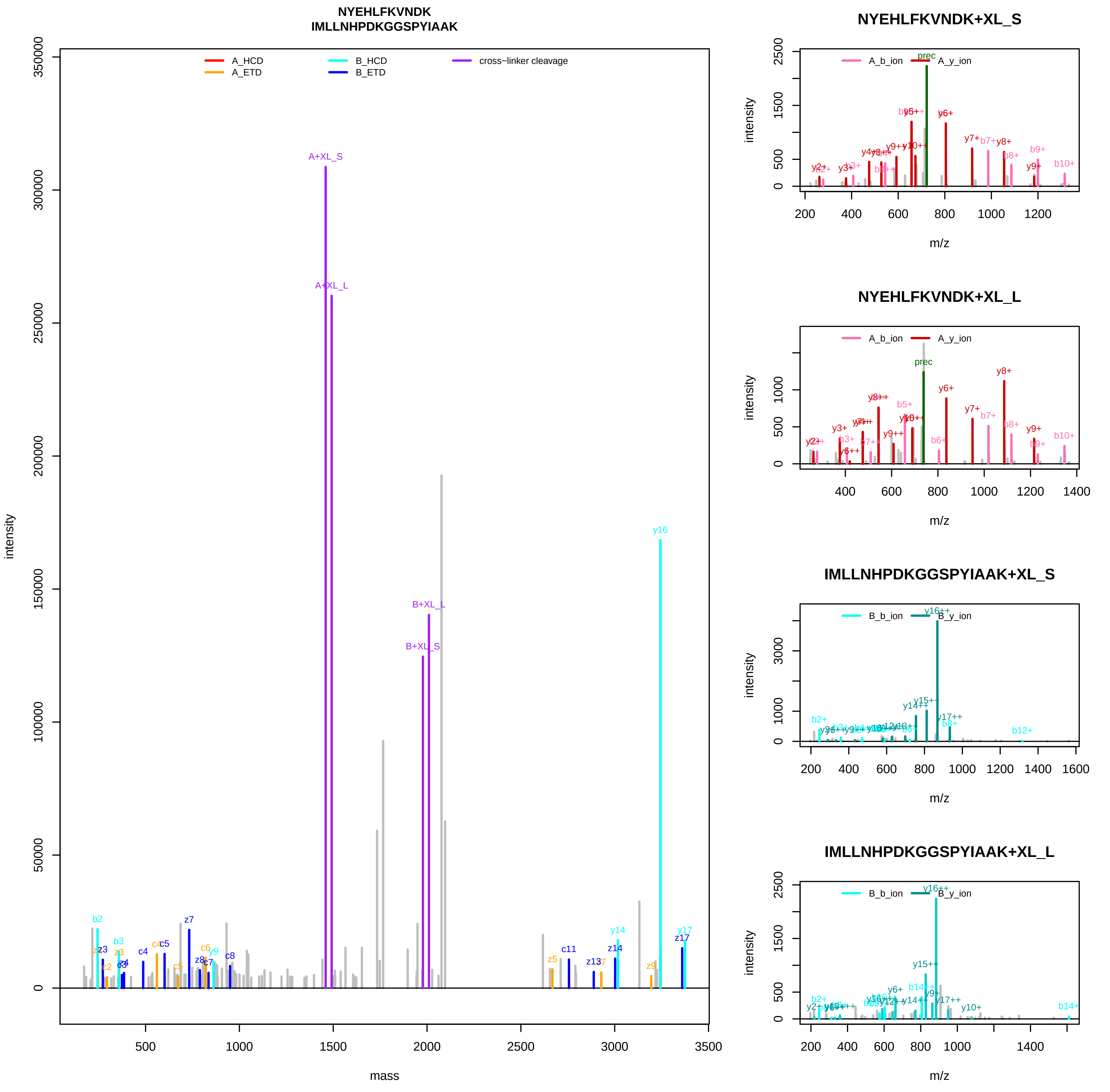

NYEHLFKVNDK
IMLLNHPDKGGSPYIAAK
A_HCD
A_ETD
B_HCD
B_ETD
cross−linker cleavage
intensity
mass
A+XL_S
A+XL_L
B+XL_L
B+XL_S
y16
y17
z17
y14
z14
z13
c11
z5
z9
b2
b3
z3
c4
c5
z7
c6
y9
c8
NYEHLFKVNDK+XL_S
NYEHLFKVNDK+XL_L
IMLLNHPDKGGSPYIAAK+XL_S
IMLLNHPDKGGSPYIAAK+XL_L
A_b_ion
A_y_ion
B_b_ion
B_y_ion
prec
m/z
y16++
y15++
y14++
y17++
b12+
b14+
y8+
y6+
y7+
b7+
b9+
b10+
